# MMP13 Expression Is Increased Following Mutant α-Synuclein Exposure and Promotes Inflammatory Responses in Microglia

**DOI:** 10.3389/fnins.2020.585544

**Published:** 2020-12-02

**Authors:** Kathryn Sánchez, Kathleen Maguire-Zeiss

**Affiliations:** ^1^Department of Biology, Georgetown University, Washington, DC, United States; ^2^Department of Neuroscience, Georgetown University Medical Center, Washington, DC, United States

**Keywords:** microglia, α-synuclein, TLRs, MMP13, MMP9, A53T

## Abstract

α-Synuclein is a 140-amino acid protein that readily misfolds and is associated with the Lewy body pathology found in sporadic and genetic forms of Parkinson's disease. We and others have shown that wild-type α-synuclein is a damage-associated molecular pattern that directly elicits a proinflammatory response in microglia through toll-like receptor activation. Here we investigated the direct effect of oligomeric mutant α-synuclein (A53T) on microglia morphology and activation. We found that misfolded A53T increased quantitative measures of amoeboid cell morphology, NFκB nuclear translocation and the expression of prototypical proinflammatory molecules. We also demonstrated that A53T increased expression of MMP13, a matrix metalloproteinase that remodels the extracellular matrix. To better understand the role of MMP13 in synucleinopathies, we further characterized the role of MMP13 in microglial signaling. We showed exposure of microglia to MMP13 induced a change in morphology and promoted the release of TNFα and MMP9. Notably, IL1β was not released indicating that the pathway involved in MMP13 activation of microglia may be different than the A53T pathway. Lastly, MMP13 increased the expression of CD68 suggesting that the lysosomal pathway might be altered by this MMP. Taken together this study shows that mutant α-synuclein directly induces a proinflammatory phenotype in microglia, which includes the expression of MMP13. In turn, MMP13 directly alters microglia supporting the need for multi-target therapies to treat Parkinson's disease patients.

## Introduction

Parkinson's disease (PD) is the second most common neurodegenerative disease after Alzheimer's disease, and affects 6.1 million people worldwide and 1% of the population over 60 years old (Ferreira and Romero-Ramos, 2018; Ray Dorsey et al., 2018). PD pathology includes the loss of dopaminergic neurons of the substantia nigra, which results in motor deficits (Braak et al., 2003; Ouchi et al., 2005; Nemani et al., 2010; Wang et al., 2015) and Lewy bodies, which are protein aggregates composed of fibrillar pathogenic α-synuclein (Giasson et al., 1999, 2002; Harding and Halliday, 2001; Mackenzie, 2001; Braak et al., 2003; Feng et al., 2010; Uversky, 2010; Vekrellis et al., 2011; Rockenstein et al., 2014; Shameli et al., 2016). Though most PD cases are sporadic, duplications, triplications, and mutations (A30P, E46K, H50Q, G51D, A53T) of the α-synuclein gene (*SNCA*) further implicate α-synuclein in disease pathogenesis (Polymeropoulos et al., 1997; Athanassiadou et al., 1999; Singleton et al., 2003; Zarranz et al., 2004; Flagmeier et al., 2016; Guella et al., 2016). These mutations in *SNCA* can influence protein conformation (Li et al., 2001; Michaluk et al., 2011; Deleersnijder et al., 2013). For example the alanine to threonine mutation (A53T), was discovered in members of the Contursi kindred and leads to faster aggregation compared to wild-type α-synuclein (Polymeropoulos et al., 1997; Athanassiadou et al., 1999; Li et al., 2001; Klein and Schlossmacher, 2006; Michaluk et al., 2011; Deleersnijder et al., 2013). Other relevant structural changes include increased beta sheet content in A53T compared to wild-type α-synuclein and a coiled filament structure (Giasson et al., 1999; Serpell et al., 2000; Coskuner and Wise-Scira, 2013). Better understanding of the pathogenic mechanisms that lead to the onset of PD can provide insight into developing targeted gene and drug therapies (Pankratz and Foroud, 2007).

In addition to Lewy bodies and nigral cell death, PD patients display increased proinflammatory cytokine levels, changes in microglial morphology, and microgliosis (Mogi et al., 1994; Blum-Degen et al., 1995; Ouchi et al., 2005; McGeer and McGeer, 2008; Gerhard, 2016). These observations emphasize the role of microglia, the innate immune cells of the brain, in PD. While microglia serve numerous functions such as synaptic pruning during development, they also respond to the central nervous system environment during infection and disease through the release of proinflammatory cytokines (Nimmerjahn et al., 2005; Paolicelli et al., 2011; Chen and Trapp, 2016; Koss et al., 2019). Investigations that utilize various conformers of α-synuclein highlight the importance of α-synuclein structure in the innate immune response, where wild-type oligomeric α-synuclein induces morphofunctional changes in microglia while the monomeric structure does not (Daniele et al., 2015; Grozdanov et al., 2019; Panicker et al., 2019).

The response of microglia to oligomeric α-synuclein is facilitated by a class of pattern recognition receptors called toll-like receptors (TLRs), which are vital signal transducers that link extracellular signals to intracellular functions and respond to damage/danger-associated molecular patterns (DAMPs), including α-synuclein (Horng et al., 2002; Béraud et al., 2011; Béraud and Maguire-Zeiss, 2012; Daniele et al., 2015; Leifer and Medvedev, 2016; Vijay, 2018). *In vitro*, wild-type α-synuclein directly binds to TLRs−1 and −2 in a Myd88-dependent manner (Daniele et al., 2015) and human PD subjects display elevated expression of TLR2 and TLR4 in peripheral monocytes and brain tissue (Watson et al., 2012; Drouin-Ouellet et al., 2015). These findings are supported by other human studies and mouse models of PD, including A53T overexpressing mice, where TLR2 promotes neuroinflammation (Kim et al., 2015; Dzamko et al., 2017). Notably, TLR activation in response to either wild-type or mutant α-synuclein leads to NFκB nuclear translocation in microglia and the subsequent release of TNFα and IL1β (Roodveldt et al., 2010; Daniele et al., 2015; Hoenen et al., 2016).

These proinflammatory cytokines also regulate matrix-metalloproteinases (MMPs); endopeptidases that are associated with α-synuclein-mediated inflammation and have been identified in patient samples (Lorenzl et al., 2004; Shubayev et al., 2006; Lee et al., 2010; He et al., 2013; Tsai et al., 2014). In the initial inflammatory response, MMPs facilitate repair and recruit phagocytic cells to clear cellular debris and apoptotic cells but if not properly regulated they become overactive and lead to neuronal damage (Fingleton, 2017). Similarly, the neurodegeneration observed in PD can be partially attributed to the dysregulation of cellular functions influenced by MMPs (Yanuck, 2019). For example, MMP1 and MMP13 increase the ability of human fibroblasts and dendritic cells to phagocytose, respectively (Perek et al., 2013; Rustenhoven et al., 2016; Hunter et al., 2018; Pelekanou et al., 2018; Yoon et al., 2020). In neurodegenerative disorders, microglial expression of MMP13 is upregulated in response to amyloid beta (Ito et al., 2007) and knockdown of MMP13 in APP/PS1E9 mice rescued learning and memory deficits (Zhu et al., 2019).

Here, we establish that MMP13 and MMP9 are released by microglia upon stimulation with mutant (A53T) oligomeric α-synuclein through the classical inflammatory pathway. Further, we characterize the direct impact of MMP13 on microglial morphology and function, providing evidence that MMP13 and A53T incite distinct inflammatory profiles, which could prove crucial in targeting primary vs. secondary inflammation.

## Materials and Methods

### Misfolding of Recombinant Human A53T

To misfold recombinant human mutant α-synuclein (A53T), lyophilized protein (Sigma-Aldrich) was resuspended in TEN buffer (10 mM Tris-HCl, pH 7.5, 1 mM EDTA, 20 mM NaCl) to a final concentration of 1 mg/mL and sonicated at 20 Hz (2 × 10 s bursts with a 10-s rest between bursts) as previously described (Béraud et al., 2011; Béraud and Maguire-Zeiss, 2012; Daniele et al., 2015). A53T was then incubated for 5 days at 37°C with rotation at 1,000 RPM, resulting in misfolded A53T. TEN buffer was incubated alongside misfolded A53T to serve as a vehicle (VEH) control for experiments.

### Silver Stain and Western Blot Analyses

Recombinant misfolded A53T (0.1 μg) underwent native polyacrylamide gradient (3–12% Bis-Tris) gel electrophoresis utilizing the NativePAGE Novex Bis-Tris gel system (Life technologies) as well as denaturing 4–20% TGX gradient gel electrophoresis using the Bio-Rad gel system. For silver stain analysis, misfolded A53T (5 μg) was subjected to denaturing gel electrophoresis as described above, and proteins were subsequently stained utilizing the Silver Stain Plus Kit according to manufacturers' instructions (Bio-Rad). For western blot analyses, proteins were then transferred to nitrocellulose membranes and fixed with 0.4% paraformaldehyde in PBS. Membranes were blocked for 1 h at room temperature in 5%TBST/NFDM [20 mM Tris-HCl pH 7.5, 150 mM NaCl, 0.1% (v/v) Tween, 5% (w/v) non-fat dry milk (NFDM)], and subsequently incubated with mouse Syn211 primary antibody (1:1000; Thermo Scientific) overnight at 4°C. Immune:antigen complexes were visualized on film (Blu-C Autoradiography Film; Stellar Scientific) following incubation with horseradish peroxidase (HRP)-conjugated goat anti-mouse 2° antibody (1:20,000; Chemicon) and Pierce Chemiluminescent Substrate (Thermo Scientific).

### Preparation of Primary Microglia

Primary microglia cultures were derived from postnatal C57/Bl6 mouse cortices (P1-3) (Daniele et al., 2014). Cortices were isolated, homogenized, and cultured for at least 16 days in Microglia Culture Media (MCM; 1x Minimal Essential Medium Earle's [MEM] supplemented with: 1 mM L-glutamine, 1 mM sodium pyruvate, 0.6% v/v D-(+)-glucose, 100 μg/ml Penicillin/Streptomycin (P/S), 4% v/v Fetal Bovine Serum (FBS), 6% v/v Horse Serum). Flasks were shaken for 3 h to isolate microglia, which were subsequently plated in Microglia Growth Media containing 5% v/v Fetal Bovine Serum (MGM; Minimum Essential Medium Earle's (MEM), supplemented with 1 mM sodium pyruvate, 0.6% (v/v) D-(+)-glucose, 1 mM L-glutamine, 100 μg/mL penicillin/streptomycin, and 5% v/v Fetal Bovine Serum) (Béraud et al., 2011, 2013; Béraud and Maguire-Zeiss, 2012; Daniele et al., 2014, 2015). For RNA extraction, cells were plated at 4.5 × 10^5^ (6-well format). For ELISAs, cells were plated at a minimum density of 3.6 × 10^4^ (24-well format). For immunocytochemistry, microglia were plated on sterile glass coverslips (12 mm; Deckglaser) at density of at least 4.0 × 10^4^ cells per well (24-well format).

### Treatment of Microglia

For the α-synuclein experiments, microglia were stimulated with MGM in 5% FBS containing 1 μg/mL misfolded A53T (Sigma-Aldrich), 20 mM TEN buffer (VEH), or LPS (100 ng/mL) for 24-h unless otherwise noted. LPS (100 ng/mL) served as a positive control where indicated. For cMMP13 experiments, cells were washed three times with MEM and subsequently treated with 20 nM cMMP13 (Enzo Life Sciences) or PBS (VEH) in MGM containing 1% FBS. Microglia were treated in a volume of 3.0 mL (6-well) or 0.5 mL MGM (24-well).

### RNA Extraction From Microglia

Following treatment with misfolded A53T or VEH, RNA was isolated from microglia utilizing a RNeasy mini Kit with on-column DNase I digestion, according to manufacturer's instructions (Qiagen). Subsequently, a NanoDrop 1000 spectrophotometer was used to determine RNA concentrations after isolation and to ensure sufficient RNA quality (Thermo Scientific) (Béraud et al., 2011, 2013; Daniele et al., 2015).

### qRT-PCR

RNA extracted from microglia (5 μg) was reverse transcribed with the High-Capacity cDNA Archive Kit (Life Technologies) in a reaction volume of 10 μL. Gene expression was quantified by qRT-PCR in a 308-well format. cDNA (1.25 μL) was combined with master mix (8.75 μL) containing the primer/probe pairs described below and TaqMan® Universal PCR Master Mix. Quantitative RT-PCR reactions were undertaken using the ABI Prism 7900HT Sequence Detection System (Life Technologies). Data were analyzed utilizing the relative quantification ΔΔCt method, normalizing target gene expression to human 18s ribosomal RNA endogenous control, followed by normalization to VEH buffer control. Primers/Probes that were used to evaluated include the following: *TNF*α Mm00443258_m1, *IL*β Mm0434228_m1, *MMP9* Mm004442991_m1, *MMP13* Mm004439491_m1, *TLR1* Mm0120884_m1, *TLR2* Mm00442346_m1, and *TLR4* Mm00445273_m1. Gene expression changes are represented as fold change (2^−ΔΔ*Ct*^). All measurements were performed with at least three biological replicates and three technical replicates (Béraud et al., 2011, 2013; Daniele et al., 2015).

### ELISAs

TNFα, IL1β, IL10, and ProMMP9 protein concentrations in conditioned media from treated microglia were quantified using an enzyme-linked immunosorbent assay (ELISA) according to the manufacturer's instructions (R&D Systems). All measurements were performed in at least three separate experiments with two biological replicates and two technical replicates (Béraud et al., 2011, 2013; Béraud and Maguire-Zeiss, 2012; Daniele et al., 2014, 2015).

### Immunocytochemistry

After treatment was stopped, cells were prepared for immunocytochemistry by washing with phosphate buffered saline (PBS) for 5 min, followed by a 20-min fixation with PBS containing 4% (w/v) paraformaldehyde and 4% (w/v) sucrose, pH 7.4 at room temperature. Cells were then permeabilized in PBS containing 0.1% (v/v) Triton X-100 for 5 min, and blocked for 1 h with PBS containing 10% (v/v) goat serum followed by overnight incubation at 4°C with rabbit anti-NFκB (1:1000; α-p65; Abcam), rabbit Iba-1 (1:750; Wako), rat CD11b (1:1000; EBT), rabbit MMP-13 (Abcam; 1:100), or rat CD68 (1:400; Bio-Rad Laboratories) in blocking buffer containing 10% goat serum. Antibody:antigen complexes were visualized following incubation with Alexa Fluor 594 or 488 conjugated goat IgG secondary antibody (1:1000) in PBS containing 0.1% (v/v) triton X-100 and 1% goat serum and subsequently counterstained with 4',6-diamidino-2-phenylindole (DAPI; 13.0 ng/μL) in PBS, followed by two 5 min PBS washes. Coverslips were mounted with Hydromount and cells were imaged and captured using a Zeiss Axioskop fluorescent microscope and AxioCam HRm camera (Carl Zeiss) (Béraud et al., 2011, 2013; Béraud and Maguire-Zeiss, 2012; Daniele et al., 2014, 2015). Images and subsequent analyses were completed by an observer blinded to treatment.

### Morphological Quantification of Microglia

Images from Iba1 immunostained microglia were captured as described above from 10 distinct, randomly selected regions of each treatment condition by a blinded observer in a serpentine pattern to ensure random representation from all areas of the coverslip. Quantification of cell shape was conducted utilizing Image J software (National Institute of Health). After images were converted to 8-bit, a uniform threshold was set to visualize either the cell body or whole cell. Each cell was manually selected to take the appropriate measurements. Cells were excluded from analysis if they were not Iba1 positive, did not have a nucleus, or had two or more nuclei. When determining the impact of α-synuclein on morphology, a total of 180 cells were analyzed for the VEH condition and 138 cells for the A53T condition. To evaluate the impact of cMMP13 on morphology, 127 cells were analyzed per condition. All measurements were performed using cells from two biological replicates (i.e., individual wells). Values from individual cells are shown and used for the statistical analysis.

### NFκB Quantification

Images from NFκB and DAPI stained microglia were captured from 5 distinct, randomly selected regions of each treatment condition by a blinded observer. Quantification of nuclear NFκB intensity was completed as previously described utilizing Image J software (National Institute of Health) (Fuseler et al., 2006; Noursadeghi et al., 2008). A total of 346 cells were analyzed from the VEH condition and 337 cells were analyzed in the A53T condition. All measurements were performed using cells from three biological replicates (i.e., individual wells). Values from individual cells are shown and used for the statistical analysis.

### Quantification of Total Fluorescence

Images from MMP13 and CD68 immunostained microglia were captured as described above from 10 distinct, randomly selected regions of each treatment condition by a blinded observer. Quantification of total fluorescence was conducted utilizing Image J software (National Institute of Health). Images were converted to 8-bit, and a threshold was subsequently set to visualize the whole cell. Each cell was manually selected to measure its total fluorescence (integrated density). For CD68 fluorescence measurements, 143 cells were analyzed in the VEH condition and 152 cells in the cMMP13 condition. For MMP fluorescence measurements, 194 cells were analyzed in the VEH condition while 171 cells were analyzed in the A53T condition. All measurements were performed using cells from three biological replicates (i.e., individual wells). Values from individual cells are shown and used for the statistical analysis.

### Statistical Analysis

All data was analyzed using GraphPad Prism, Version 8. Student's *t*-test was used to analyze data from qRT-PCR, ELISAs, morphological measurements, nuclear fluorescent intensity, and total fluorescent intensity unless otherwise stated. Data points shown represent individual cells for morphology and fluorescent intensity and values were not averaged per well since wells are not always homogenous in primary cultures (Allen et al., 2016; Hoenen et al., 2016; De Biase et al., 2017; Winland et al., 2017; Abdolhoseini et al., 2019; Svoboda et al., 2019). Significance threshold was set at *P* ≤ 0.05. Data are represented as mean ± SEM.

## Results

### Characterization of Misfolded A53T α-Synuclein

**Figure 1:**
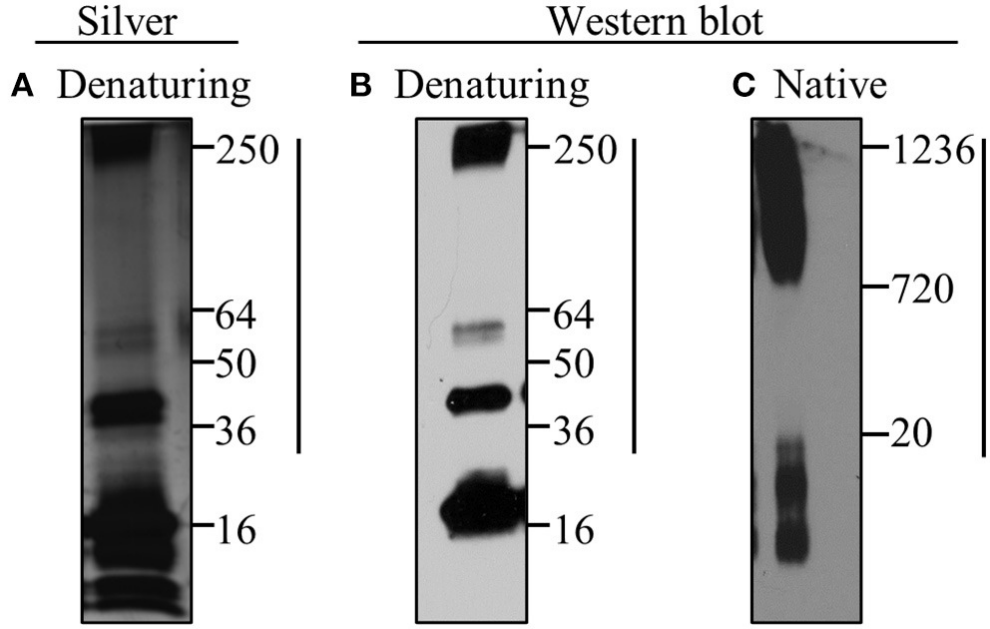
Misfolded A53T contains α-synuclein oligomers. Misfolding of recombinant A53T was induced by subjecting the protein to 37°C and rotation. Misfolded A53T was subjected to **(A)** silver stain and **(B,C)** western blot analyses with the human specific α-synuclein antibody (Syn211; Thermo Scientific; 1:1000) following **(A,B)** denaturing and **(C)** native gel electrophoresis. A53T monomer is ~16kDa and larger oligomeric conformers are indicated by the vertical line. Molecular weight markers in kDa are indicated to the right in each panel.

In order to investigate the effect of mutant α-synuclein on microglia, recombinant human A53T α-synuclein (A53T) was misfolded using standard conditions and characterized for the presence of oligomers (Béraud and Maguire-Zeiss, 2012; Béraud et al., 2013; Daniele et al., 2015). As shown in Figure 1, under denaturing gel electrophoresis conditions, the misfolded recombinant A53T contained both monomeric and higher molecular weight oligomeric species (36–250 kDa; Figure 1A: silver stain analysis; Figure 1B: α-synuclein western blot analysis). Under non-denaturing (native) western blot conditions, the majority of the A53T conformers were large molecular weight species between 720 and 1,236 kDa (Figure 1C). These analyses confirmed the presence of α-synuclein oligomers in the misfolded A53T along with small molecular weight species as previously reported (de Oliveira and Silva, 2019).

### Microglial Exposure to A53T Results in Morphological Changes

**Figure 2:**
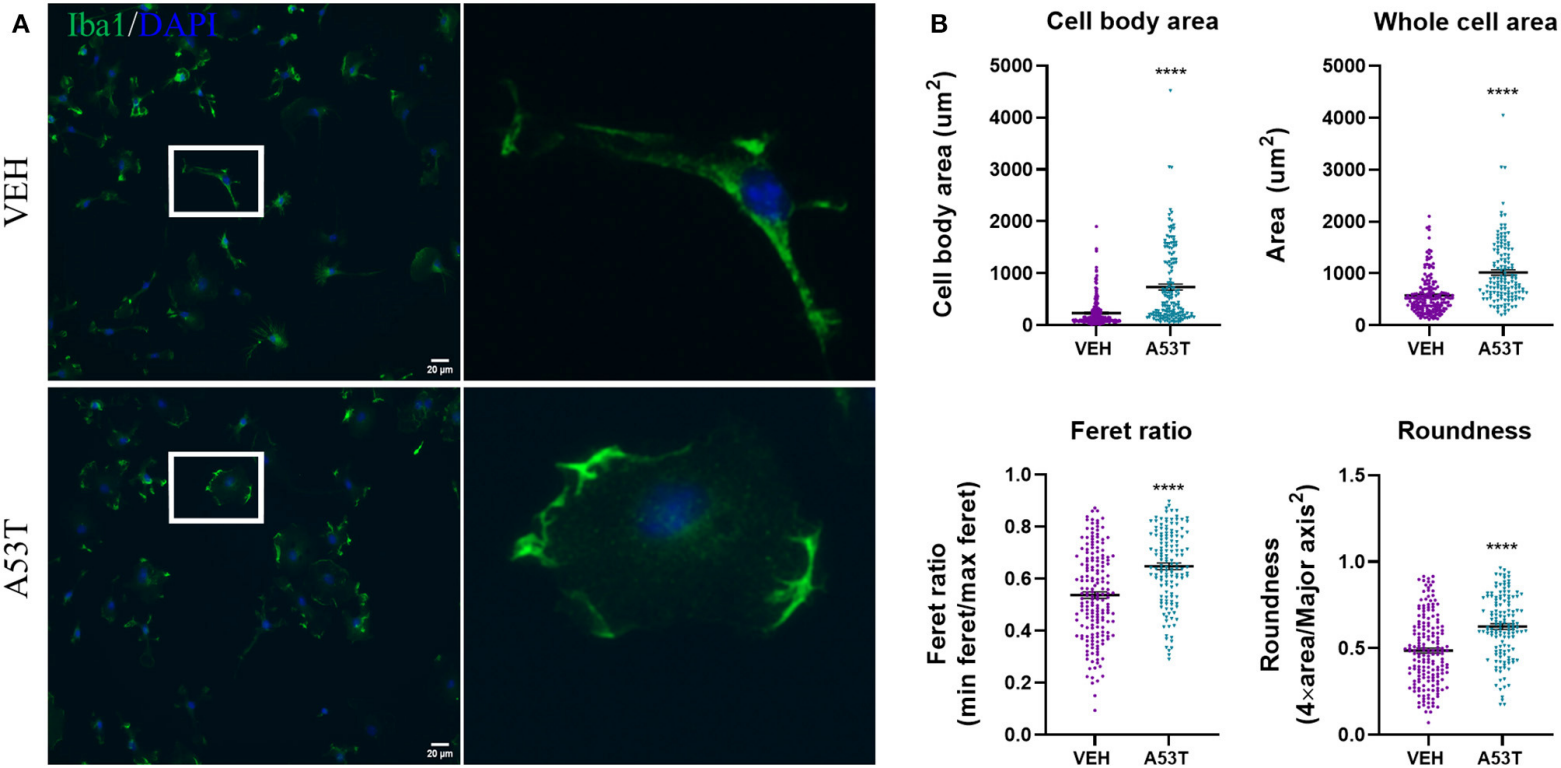
A53T alters microglial morphology. **(A)** Microglia were treated with 1 μg/mL misfolded A53T or vehicle (VEH) for 24 h and Iba1 immunocytochemistry (green) was used to visualize the cells. Nuclei were counterstained with DAPI (blue). Enlarged microglia (white box) are shown to the right. **(B)** Consistent with a visual change, A53T exposure led to a change in morphological parameters (cell body area, whole cell area, Feret ratio, and roundness) compared to vehicle treatment; VEH *n* = 180 cells and A53T *n* = 138 cells. Values are reported as mean ± SEM, using an unpaired Student's *t*-test; ****P ≤ 0.0001.

Knowing that oligomeric species were present in the A53T α-synuclein, we sought to evaluate the effect of A53T (1 μg/mL) on the morphology of primary microglia derived from mouse cortices, 24 h post-exposure. Quantification of microglial morphology was conducted using ImageJ analyses following immunocytochemistry for Iba1, a calcium-binding adapter protein expressed by microglia (Figure 2A). A53T exposure induced a statistically significant change in measurements that are associated with a shift from ramified or rod-like morphologies to an amoeboid shape (Figure 2B). For example, cell body cell area increased from 233.7 um^2^ in vehicle-exposed microglia to 736.6 um^2^ after A53T stimulation (*P* ≤ 0.0001; Figure 2B). Whole cell area was also enlarged following A53T exposure (VEH = 572 um^2^; A53T = 1,017 um^2^; *P* ≤ 0.0001; Figure 2B). The Feret (aspect) ratio was lower in vehicle-treated microglia (0.54) indicating a more elongated shape compared with A53T-stimulated cells (0.65; *P* ≤ 0.0001; Figure 2B). Likewise, roundness (the inverse of the Feret ratio; where a circle has a roundness value of 0.5), was 0.49 after vehicle exposure compared with 0.62 in A53T-exposed microglia (*P* ≤ 0.0001; Figure 2B). Taken together, these measurements demonstrated that A53T exposure led to a shift in microglia morphology from a rod-like shape to a more amoeboid shape, which is often associated with a rise in phagocytosis (Perez-Pouchoulen et al., 2015; Winland et al., 2017; Dubbelaar et al., 2018).

### Toll Like Receptors (TLRs) Are Increased in the Response of Microglia to Mutant α-Synuclein

**Figure 3:**
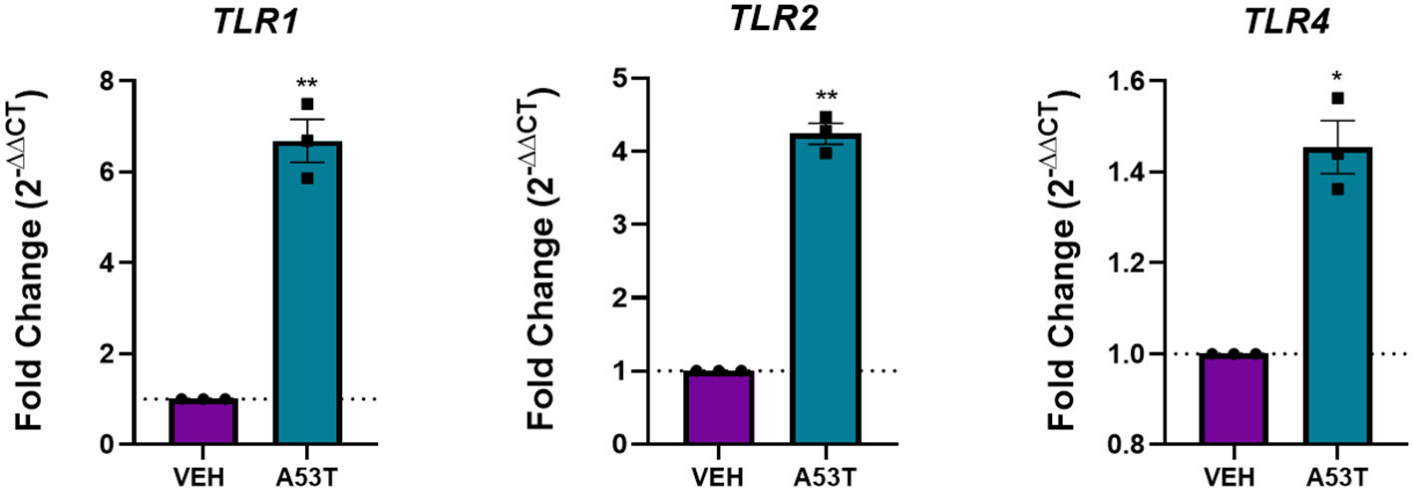
TLRs are upregulated following A53T treatment of microglia. Microglia were treated with 1 μg/mL misfolded A53T or vehicle (VEH) for 24 h and changes in gene expression for *TLR 1, 2*, and *4* were evaluated using qRT-PCR. Microglia stimulated with misfolded A53T displayed increased levels of *TLR1, TLR2*, and *TLR4* mRNA expression compared to vehicle, (*n* = 3 wells/treatment). Values are reported as mean ± SEM, using an unpaired Student's *t*-test; **P ≤ 0.01 and *P ≤ 0.05.

Since microglia morphology is associated with different activation states and receptor signaling pathways, we next sought to identify the receptors that could be involved in the response of microglia to mutant α-synuclein. Pattern recognition receptors (PRRs) are important signal transducers linking extracellular (DAMPS) to intracellular functions. Furthermore, a subclass of PRRs, TLRs, is associated with microglial responses to oligomeric wild-type α-synuclein (Béraud and Maguire-Zeiss, 2012; Béraud et al., 2013; Daniele et al., 2015). Previous work from our laboratory and others provides evidence that TLR1 and TLR2, well-characterized PRRs present on the microglia cell membrane, are upregulated upon α-synuclein stimulation and bind α-synuclein directly (Daniele et al., 2015). Furthermore, previous evidence has also implicated a role for TLR4 in α-synuclein-mediated signaling, making it important to investigate (Fellner et al., 2013). To determine if TLRs are affected by exposure to mutant α-synuclein, TLR mRNA expression was evaluated 24 h following A53T (1 μg/mL) stimulation of microglia. Here we show elevated expression of *TLR1* (6.7-fold; *P* ≤ 0.01), *TLR2* (4.2-fold; *P* ≤ 0.01), and *TLR4* (1.5-fold; *P* ≤ 0.05) after A53T exposure compared with vehicle-treated cells (Figure 3). These findings suggest that mutant α-synuclein signals through microglial TLRs.

### A53T Induces NFκB Nuclear Translocation Followed by mRNA Expression and Release of Proinflammatory Cytokines

**Figure 4:**
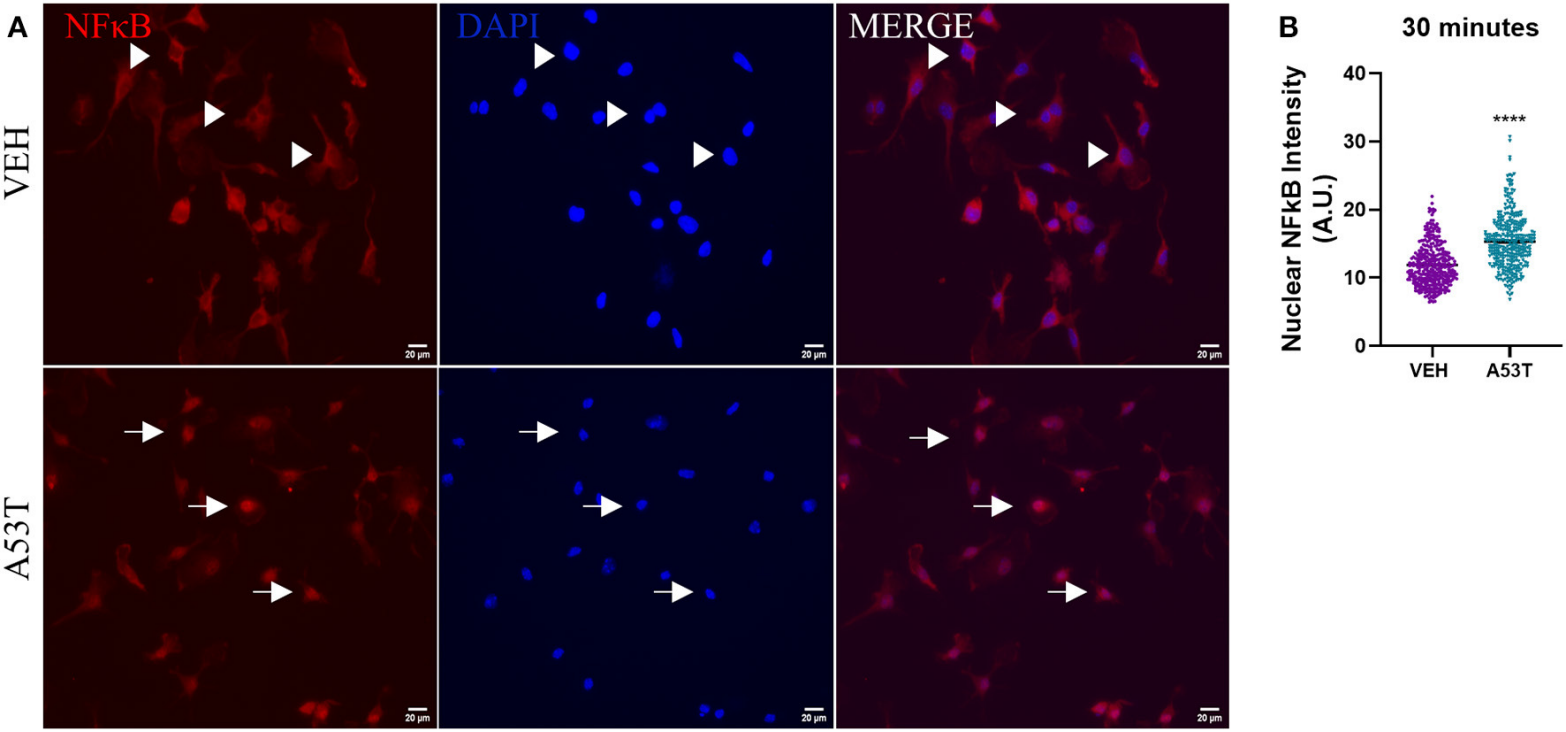
NκFB nuclear translocation is increased in microglia stimulated by misfolded A53T. Nuclear translocation of NFκB was analyzed following a 30-min stimulation with 1 μg/mL misfolded A53T or vehicle (VEH). **(A)** The presence of cytoplasmic (arrowheads) and nuclear (arrows) NFκB (red) following immunocytochemistry (nuclei; DAPI, blue). **(B)** Nuclear translocation of NFκB was quantified and shown to be significantly elevated in A53T-stimulated microglia compared to vehicle treatment; VEH *n* = 346 cells and A53T *n* = 337 cells. Values are reported as mean ± SEM, using an unpaired Student's *t*-test; ****P ≤ 0.0001.

**Figure 5:**
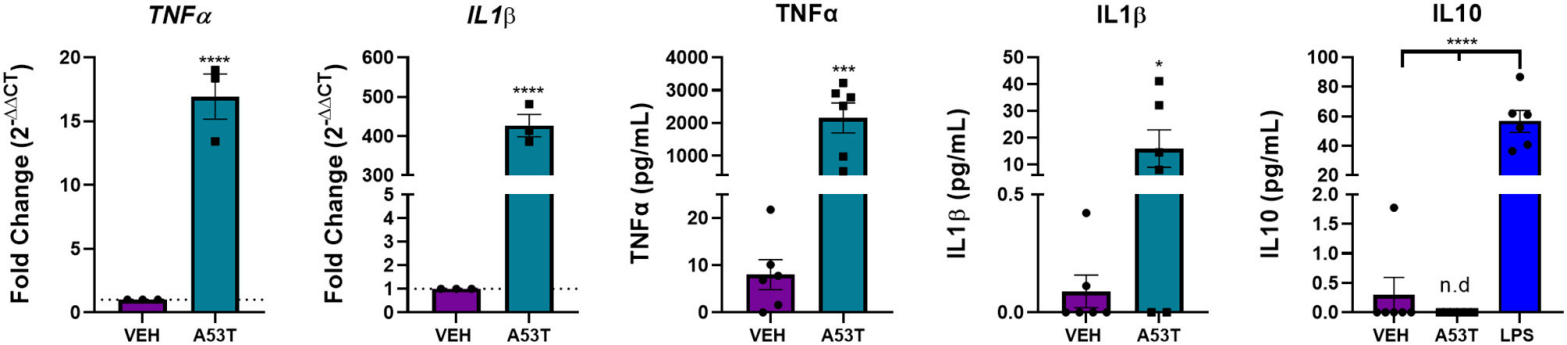
The expression and release of proinflammatory cytokines are upregulated in A53T-exposed microglia. Microglia were exposed to 1 μg/mL misfolded A53T or vehicle (VEH) for 24 h. A53T-exposed microglia display increased gene expression of the classical proinflammatory cytokines, *TNF*α and *IL1*β compared to VEH treated cells (*n* = 3 wells/treatment). TNFα and ILIβ release was also increased following A53T exposure (ELISA; *n* = 6 wells/treatment). IL10, an anti-inflammatory cytokine, was not released in response to A53T, but was detected following treatment with 100 ng/mL LPS (*n* = 6 wells/treatment); one-way ANOVA with Dunnett's *post-hoc*. Other ELISAs are reported as mean ± SEM, using an unpaired Student's *t*-test; ****P ≤ 0.0001, ***P ≤ 0.001, and *P ≤ 0.05.

After determining that *TLR* expression was upregulated in response to mutant α-synuclein, we sought to investigate the downstream signaling pathways involved in the microglial response to A53T. We focused on NFκB nuclear translocation, which is downstream of TLR1, TLR2, and TLR4 activation. Using A53T (1 μg/mL), we demonstrated a 1.3-fold increase in NFκB nuclear translocation 30 min after A53T exposure of microglia compared with vehicle-treated cells (*P* ≤ 0.0001; Figure 4). Since NFκB regulates the expression of several proinflammatory molecules, we next sought to determine changes in mRNA expression and protein release of the prototypical proinflammatory cytokines, TNFα and IL1β. Twenty-four hours post-A53T (1 μg/mL) exposure, microglia displayed a statistically significant 16.9-fold increase in *TNF*α gene expression (*P* ≤ 0.0001) and a 426.9-fold increase in *IL1*β gene expression compared with vehicle-treated cells (*P* ≤ 0.0001; Figure 5). In line with an increase in mRNA expression, TNFα and IL1β protein release were significantly elevated in conditioned media after A53T exposure compared with vehicle-treated microglia (TNFα: A53T = 2152.27 pg/mL; VEH = 8.03 pg/mL, *P* ≤ 0.001; IL1β: A53T = 15.95 pg/mL; VEH = 0.09 pg/mL, *P* ≤ 0.05; Figure 5).

We next interrogated the effect of A53T on the anti-inflammatory cytokine, interleukin 10 (IL10). In our hands, there was no significant IL10 release following A53T exposure of microglia (Figure 5). As a positive control, the primary microglia responded as previously reported to bacterial lipopolysaccharide (LPS) with an induction of IL10 (Figure 5; *P* ≤ 0.0001) (Ledeboer et al., 2002; Lively and Schlichter, 2018). An interaction between proinflammatory and anti-inflammatory molecules is crucial to maintain microglial homeostasis, and our findings suggest this balance is disrupted by mutant α-synuclein.

### A53T Enhances the Expression of Microglial-Derived MMPs

Matrix metalloproteinases (MMPs) are a family of endopeptidases released by glia and neurons in response to inflammation (Choi et al., 2008; Béraud and Maguire-Zeiss, 2012; Könnecke and Bechmann, 2013; Allen et al., 2016; Bozzelli et al., 2019). Since the expression of a subset of MMPs is also regulated by NFκB nuclear translocation, we next asked whether mutant α-synuclein affects the mRNA and protein levels of MMP9, MMP13, and MMP3. We chose these MMPs since NFκB regulates MMP9 and MMP13 expression, and in turn and MMP13 can continue to regulate MMP9 activity (Bond et al., 1999; Chase et al., 2002; Toriseva and Kähäri, 2009; Li et al., 2012). Lastly, we chose MMP3 due to its well-established link to PD pathology and neuroinflammation (Lee et al., 2010; Choi et al., 2011; Yoon et al., 2020).

**Figure 6:**
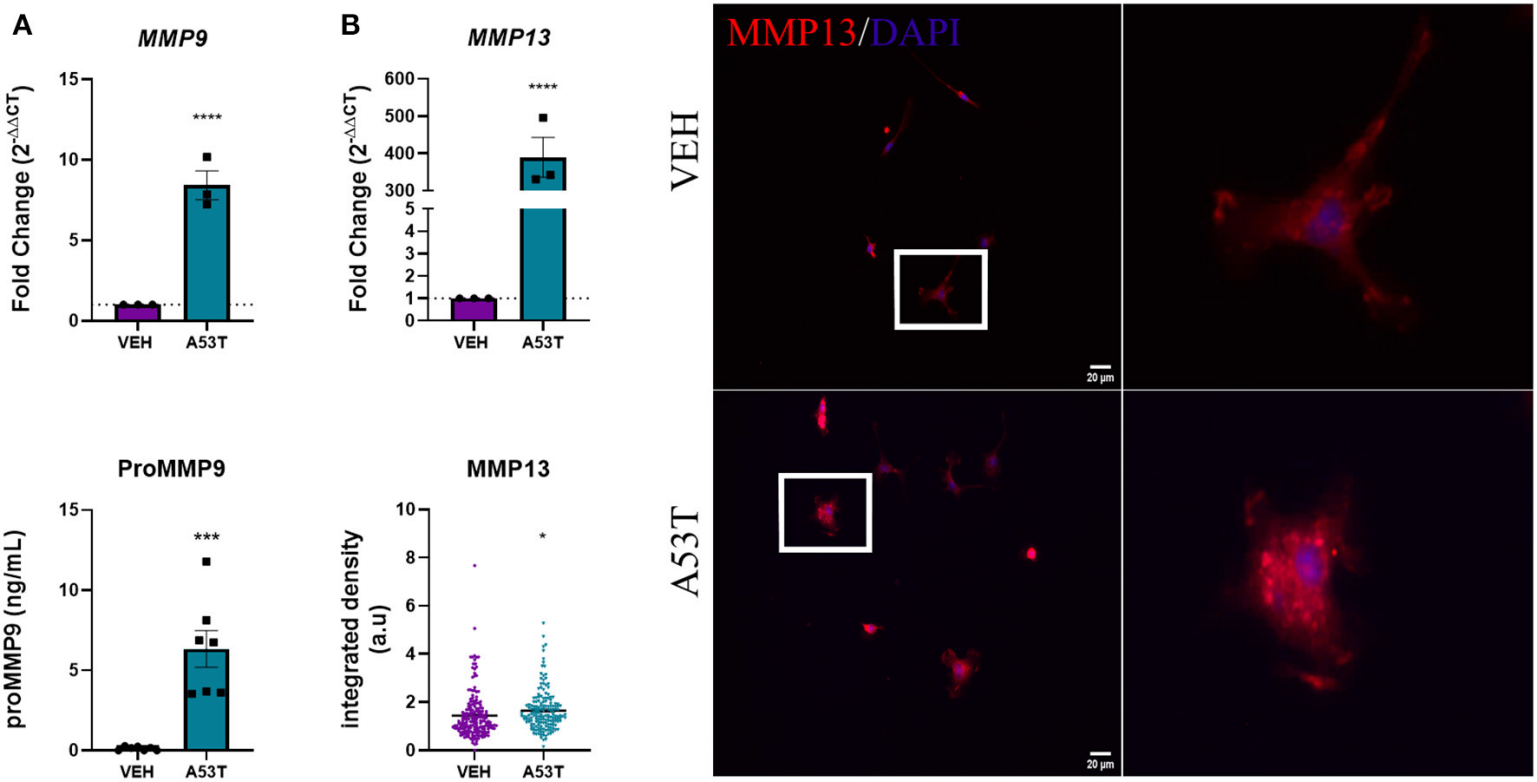
Microglia-derived MMPs are upregulated in response to A53T. Microglia were exposed to 1 μg/mL misfolded A53T or vehicle (VEH) for 24 h and evaluated for changes in MMP expression and release. **(A)** A53T exposure upregulated *MMP9* expression (*n* = 3 wells/treatment) and protein release (*n* = 7 wells/treatment) compared to vehicle-treated cells. **(B)** A53T exposure increased *MMP13* mRNA expression and MMP13 protein (integrated density following MMP13 immunocytochemistry red; DAPI, blue) compared to vehicle treatment (VEH *n* = 194 cells and A53T *n* = 171 cells). Values are reported as mean ± SEM, using an unpaired Student's *t*-test; ****P ≤ 0.0001, ***P ≤ 0.001, and *P ≤ 0.05.

Here we show that, A53T exposure (1 μg/mL) led to an 8.42-fold increase in *MMP9* gene expression compared with vehicle-treated microglia (*P* ≤ 0.0001; Figure 6A). Furthermore, microglial release of MMP9 protein was significantly increased in A53T-treated microglia compared with vehicle-treated cells (A53T: 6.33 ng/mL proMMP9; VEH: 0.12 ng/mL; *P* ≤ 0.001; Figure 6A). *MMP13* mRNA expression was also significantly upregulated upon A53T stimulation (389.68-fold increase compared with vehicle; *P* ≤ 0.0001; Figure 6B). Immunocytochemistry analysis also revealed a statistically significant elevation in MMP13 protein expression in A53T-exposed microglia compared with vehicle-treated cells (*P* ≤ 0.05; Figure 6B). While an elevation in MMP9 and MMP13 were apparent, MMP3 release was undetected, though the control recombinant MMP3 was detected (data not shown). These findings demonstrate that A53T incites a complex proinflammatory morphofunctional phenotype in microglia, increasing the expression of both cytokines and a subset of MMPs.

### cMMP13 Directly Affects Microglia Structure and Function

**Figure 7:**
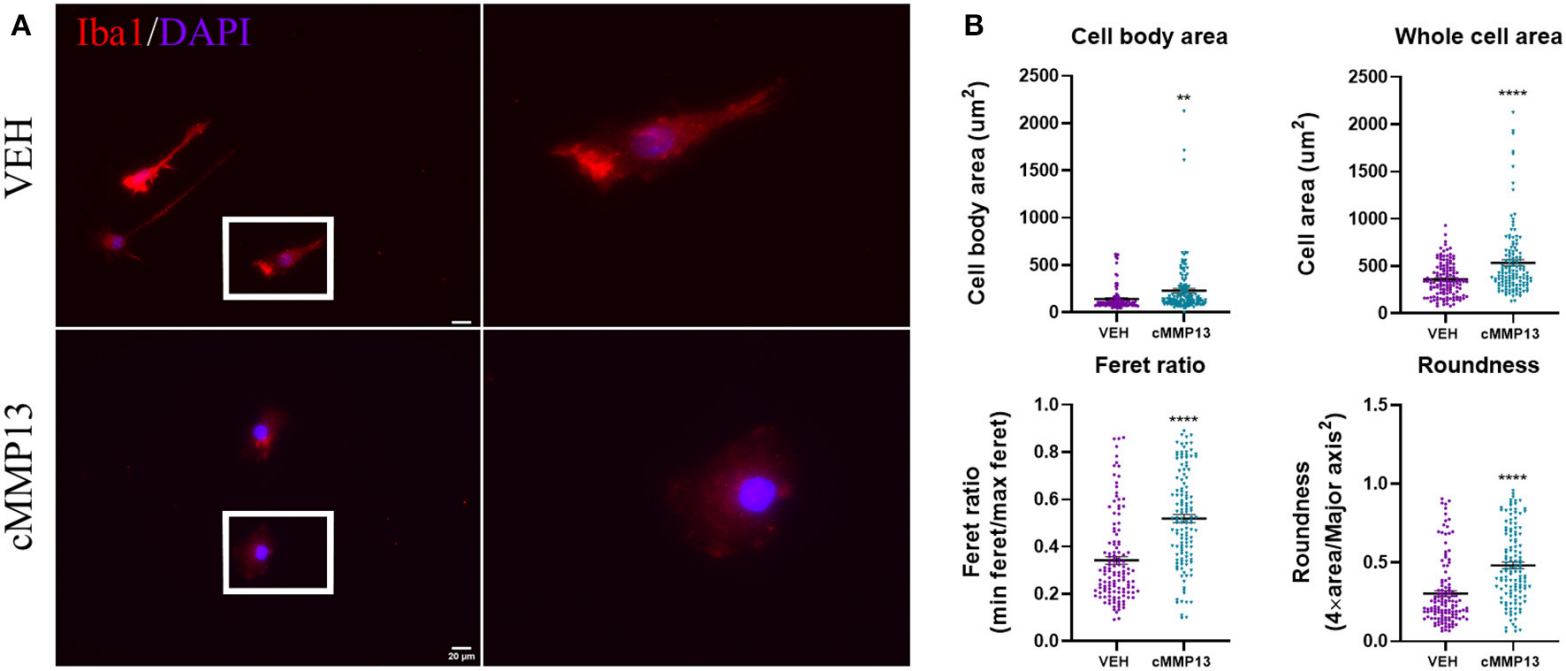
cMMP13-stimulated microglia exhibit an amoeboid morphology. **(A)** Microglia were stimulated with 20 nM cMMP13 for 24 h and subsequently evaluated for morphological changes using Iba1 immunocytochemistry (red). Nuclei were counterstained with DAPI (blue). **(B)** Cell body area, whole cell area, Feret ratio, and roundness were quantified using ImageJ analyses. cMMP13 exposure changed the morphological parameters indicative of a switch from a rod-like (vehicle treatment) to amoeboid morphology, *n* = 127 cells for both cMMP13 and VEH. Values are reported as mean ± SEM, using an unpaired Student's *t*-test; **P ≤ 0.01 and ****P ≤ 0.0001.

After establishing that A53T elevated MMP13 levels, we next asked whether MMP13 itself alters microglial function. To this end, microglia were exposed to recombinant catalytic MMP13 (cMMP13; 20 nM) for 24 h and evaluated for morphological changes using Iba1 immunocytochemistry and ImageJ analyses as described above (Figure 7A). cMMP13 induced a statistically significant change in all morphometric measurements (Figure 7B). Specifically, cell body area increased from an average of 141 um^2^ in vehicle-stimulated cells to 229.5 um^2^ following cMMP13 exposure (*P* ≤ 0.01; Figure 7B). Whole cell area was also increased with cMMP13 treatment (VEH = 358.3 um^2^; cMMP13 = 535.3 um^2^; *P* ≤ 0.0001; Figure 7B). Lastly, the changes in morphological parameters indicate a shift from a rod-like shape to a more circular microglial phenotype. For example, the Feret ratio displayed by vehicle-treated cells was 0.34 compared with 0.52 following cMMP13 exposure (*P* ≤ 0.0001; Figure 7B) and roundness increased from 0.3 in vehicle-treated cells to 0.48 in cMMP13-stimulated microglia (*P* ≤ 0.0001; Figure 7B). Combined, these changes in shape suggest a shift in cMMP13-stimulated microglia to a more amoeboid morphology and this could be indicative of functional changes in the microglia.

**Figure 8:**
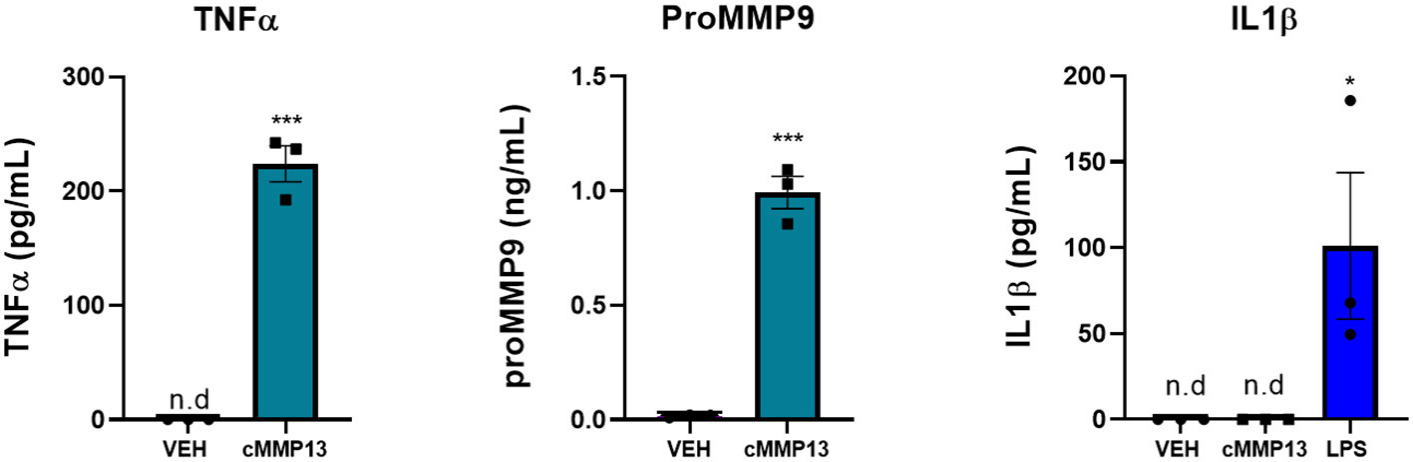
The expression of a subset of microglia-derived proinflammatory molecules is increased following cMMP13 treatment. ELISAs were performed on microglia-conditioned media after cMMP13 or VEH exposure. Microglia treated with 20 nM cMMP13 for 24 h increased the release of TNFα and MMP9, but not IL1β (*n* = 3 wells/treatment). 100 ng/mL LPS treatment increased IL1β release as expected; One-way ANOVA with Dunnett's *post-hoc* (*n* = 3 wells/treatment). Other ELISAs are reported as mean ± SEM, using an unpaired Student's *t*-test; ***P ≤ 0.001 and *P ≤ 0.05.

To determine whether the cMMP13-induced changes in microglia morphology reflect an altered proinflammatory state, we investigated the release of proinflammatory molecules following treatment. Twenty-four hours post-exposure, cMMP13-treated microglia released significantly more TNFα (223.85 pg/mL, *P* ≤ 0.001; Figure 8) than vehicle-treated cells (not detected). However, cMMP13-exposed microglia did not release detectable levels of IL1β (Figure 8). Microglia were capable of IL1β release as demonstrated following LPS-stimulation (*P* ≤ 0.05; Figure 8) and A53T exposure (Figure 5).

Since MMP13 regulates the expression of MMP9 in other cell types, we next asked whether MMP13 stimulates the release of MMP9 in the microglia (Knäuper et al., 1997; Lausch et al., 2009). To that end, MMP9 release was quantified 24-h post-cMMP13 treatment and found to be increased nearly 50-fold compared with vehicle-treated microglia (cMMP13 = 0.99 ng/mL; VEH = 0.02 ng/mL; *P* ≤ 0.001; Figure 8).

**Figure 9:**
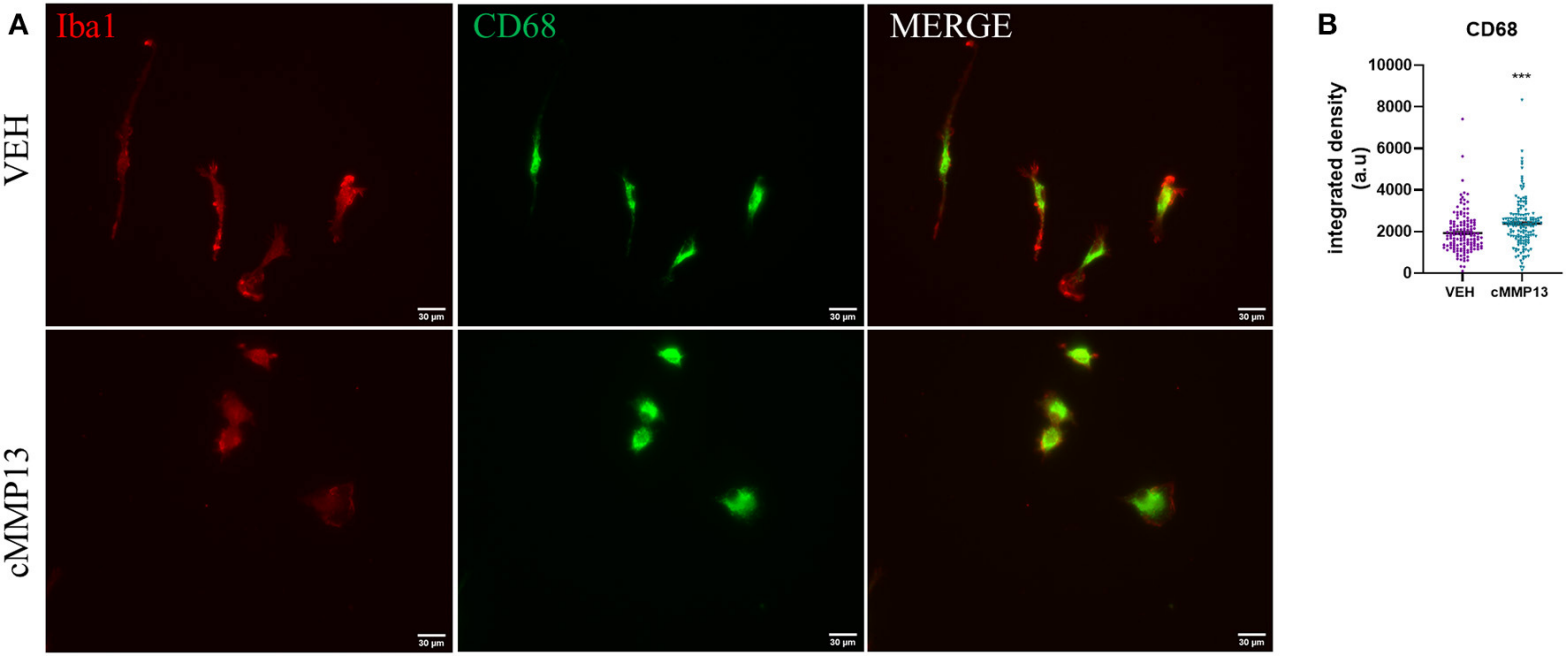
cMMP13-stimulated microglia increased the lysosomal marker, CD68. **(A)** CD68 (green) and Iba1 (red) immunofluorescence was conducted after 24 h of 20 nM cMMP13 or vehicle treatment of microglia. **(B)** CD68 total fluorescent signal was elevated in cMMP13-treated microglia; *n* = 143 VEH cells and *n* = 152 cMMP13 cells. Values are reported as mean ± SEM, using an unpaired Student's *t*-test; ***P ≤ 0.001.

Since inflammation increases lysosomal exocytosis, which subsequently leads to the release of MMPs (Figure 6), we probed for changes in CD68, a glycoprotein localized to the endosomal and lysosomal compartment (Figure 9A) (Monif et al., 2016; Xia et al., 2019). Twenty-four hours after cMMP13 treatment, there was a statistically significant elevation in total CD68 compared with vehicle-exposed cells (*P* ≤ 0.001; Figure 9B). These findings support that the inflammatory phenotype induced by cMMP13 alters microglial structure and function including endosomal/lysosomal exocytosis.

## Discussion

Through this investigation we characterized critical aspects of the inflammatory profile elicited by mutant α-synuclein. Importantly, we used high molecular weight oligomeric α-synuclein conformers, confirmed by silver stain and western blot analyses, relevant to PD pathology, and necessary for microglial activation (Sharon et al., 2003; Tofaris et al., 2003; Roodveldt et al., 2010; Daniele et al., 2015; Hoenen et al., 2016). We also discovered the novel involvement of matrix-metalloproteinase 13 (MMP13) in α-synuclein-mediated inflammation and identified its role in facilitating microglial signaling and lysosomal exocytosis. Our findings that microglia display different inflammatory profiles after A53T exposure compared with cMMP13 stimulation support the hypothesis that there are unique differences in the microglia response to these proteins. Moreover, our results implicate MMPs in sustaining inflammation and modifying microglia phenotype, which is crucial to the chronic activation state that is sustained during disease (McGeer et al., 1988; McGeer and McGeer, 2008).

Here, we established that α-synuclein stimulation of microglia led to morphofunctional changes through a classical inflammatory pathway profile. Furthermore, while we previously showed that oligomeric wild-type α-synuclein stimulates IL10 release, here in agreement with Roodveldt et al. we did not detect IL10 following A53T exposure (Roodveldt et al., 2010; Daniele et al., 2015). Given that cytokine equilibrium is crucial to microglial homeostasis, it is important to consider how IL10 mediates inflammation through the regulation of cytokines such as TNFα (Wang et al., 1995; Shin et al., 1999; Sato et al., 2003). For example, this absence of IL10 may explain the higher levels of proinflammatory cytokines observed in A53T-exposed microglia (Hoenen et al., 2016).

Like proinflammatory cytokines, MMPs are also linked to PD and are an integral part of the inflammatory response observed in microglia (Lorenzl et al., 2002; Kim et al., 2007; Lee et al., 2010; Choi et al., 2011; He et al., 2013; Annese et al., 2015). Here, we explored specific MMPs, and found an increase in the MMP9 and MMP13 but not MMP3 after A53T exposure. The absence of MMP3 could be important to familial patient pathology in particular since clinically MMP3 has only been found in sporadic cases (Choi et al., 2011). Furthermore, in preliminary work, we found that wild-type α-synuclein also induces MMP13 mRNA expression in microglia with no statistically significant differences between the two variants of α-synuclein (data not shown), suggesting the effect on microglia might have broader implications that include sporadic PD.

After establishing the upregulation of MMP13 expression as a result of α-synuclein exposure, our study further interrogated the direct effect of this metalloproteinase on microglia since it induces an inflammatory response in astrocytes (Bozzelli et al., 2019). To that end, we showed for the first time that cMMP13 initiated morphofunctional changes in microglia typically seen during an inflammatory insult. We reported a shift in microglial morphology that occurred in conjunction with the release of the proinflammatory molecules TNFα and MMP9. These results suggest that MMPs contribute to inflammation by also promoting the release of proinflammatory proteins such as TNFα and MMP9 (Wang et al., 2015). MMPs could contribute to the neurodegeneration through these inflammatory molecules since TNFα stimulates neuronal apoptosis in the substantia nigra while MMP9 alters neuron structural integrity (McCoy et al., 2006; Jara et al., 2007; Michaluk et al., 2011; Murase et al., 2016; Wiera et al., 2017). Overall, the release of these inflammatory molecules could further perpetuate the vicious cycle of inflammation often seen in neurodegenerative diseases where dendritic spine loss is observed (McNeill et al., 1988; Anglade et al., 1996; Stephens et al., 2005; Smith et al., 2009). We also demonstrated that IL1β was differentially released, as it was upregulated following A53T but not cMMP13 treatment of microglia. Though this finding needs to be further examined, our work implies that inflammasome formation may be triggered by A53T but not cMMP13. Furthermore, while in the past microglial activation has been classified as M1-like or M2-like, it is now widely accepted that activated microglia display complex expression profiles, a unique combination of both proinflammatory and anti-inflammatory molecules (Chen and Trapp, 2016).

The complexity of the response of microglia to disease associated molecular pathogens is likely in part due to the microenvironment and inherent cellular heterogeneity. In this work when individual cell responses to either A53T or MMP13 were interrogated we analyzed each cell as an independent observation. We chose this method of analysis since primary microglia and microglia *in situ* display cellular heterogeneity (De Biase et al., 2017; Abdolhoseini et al., 2019). In fact, when an analysis was conducted in which each well was considered an independent observation, some morphological measurements [cell body area (Figure 2B; *P* = 0.06 and Figure 7B; *P* = 0.24), whole cell area (Figure 7B; *P* = 0.09)] and the integrated fluorescence intensity of MMP13 (Figure 6B; *P* = 0.6) and CD68 (Figure 8B; *P* = 0.08) no longer met statistical significance. This suggests that some of the microglial responses were more robust on a population basis than others.

However, taken together, the results presented here provide evidence that familial forms of PD may be better managed using a multitherapeutic approach targeting TLRs as well as MMPs (Caplan and Maguire-Zeiss, 2018). Using TLR2 knockout mice, Kim et al. show that loss of TLR2 signaling attenuates α-synuclein-mediated inflammation and previous work from our lab shows that attenuation of TLR1/2 signaling decreases wild-type α-synuclein-mediated microglial activation (Daniele et al., 2015; Kim et al., 2015). Drugs known to inhibit the NFκB pathway also warrant further investigation as do clinically relevant MMP inhibitors (Allen et al., 2016; Bozzelli et al., 2019; Giriwono et al., 2019). While we have better characterized specific MMPs and TLRs in familial PD, the implications of MMP13 on microglia phagocytosis and process motility are yet to be understood even though we show here that CD68 expression was increased. One possible MMP13 target, which is the focus of future research, is protease receptor-1, a GPCR linked to enhanced neuroinflammation, which is activated by MMP-directed cleavage (Suo et al., 2002; Brkic et al., 2015; Allen et al., 2016; Bozzelli et al., 2019). The role of MMP13 in human disease specifically requires the use of human tissue human microglia derived from iPSCs, and improved PET imaging. Taking these future directions would allow for a better understanding of possible multiple therapeutic approaches that could be used in PD.

## Data Availability Statement

The original contributions presented in the study are included in the article/Supplementary Material, further inquiries can be directed to the corresponding author/s.

## Author Contributions

KS and KM-Z designed the experiments. KS performed experiments and wrote the first draft of the manuscript. Both authors analyzed data, wrote sections of the manuscript, contributed to manuscript revision and read, and approved the submitted version.

## Conflict of Interest

The authors declare that the research was conducted in the absence of any commercial or financial relationships that could be construed as a potential conflict of interest.
