# Supplementary material for: MMP13 Expression Is Increased Following Mutant α-Synuclein Exposure and Promotes Inflammatory Responses in Microglia
Source: Front Neurosci. 2020 Dec 2;14:585544. doi: 10.3389/fnins.2020.585544 (PMC7738560; doi:10.3389/fnins.2020.585544)

Supplementary Material

1 Supplementary Figure 1.

(A) 0.1ug of samples containing A53T synuclein or wild-type (WT) synuclein were subjected to denaturing gel electrophoresis as described in Figure 1. (B) 0.1ug of A53T protein was also subjected to native gel electrophoresis as described in Figure 1. Two lanes of each variant were loaded in each gel. To ensure the purity of the recombinant protein, a silver stain was also conducted (C). Note from left to right, lane 1 is molecular weight markers, lanes 2-3 wild-type (WT) synuclein, lanes 4-5 A53T synuclein, and lanes 6-10 indicated by the horizontal line and “X” indicates samples that are unrelated to the α-synuclein analysis.

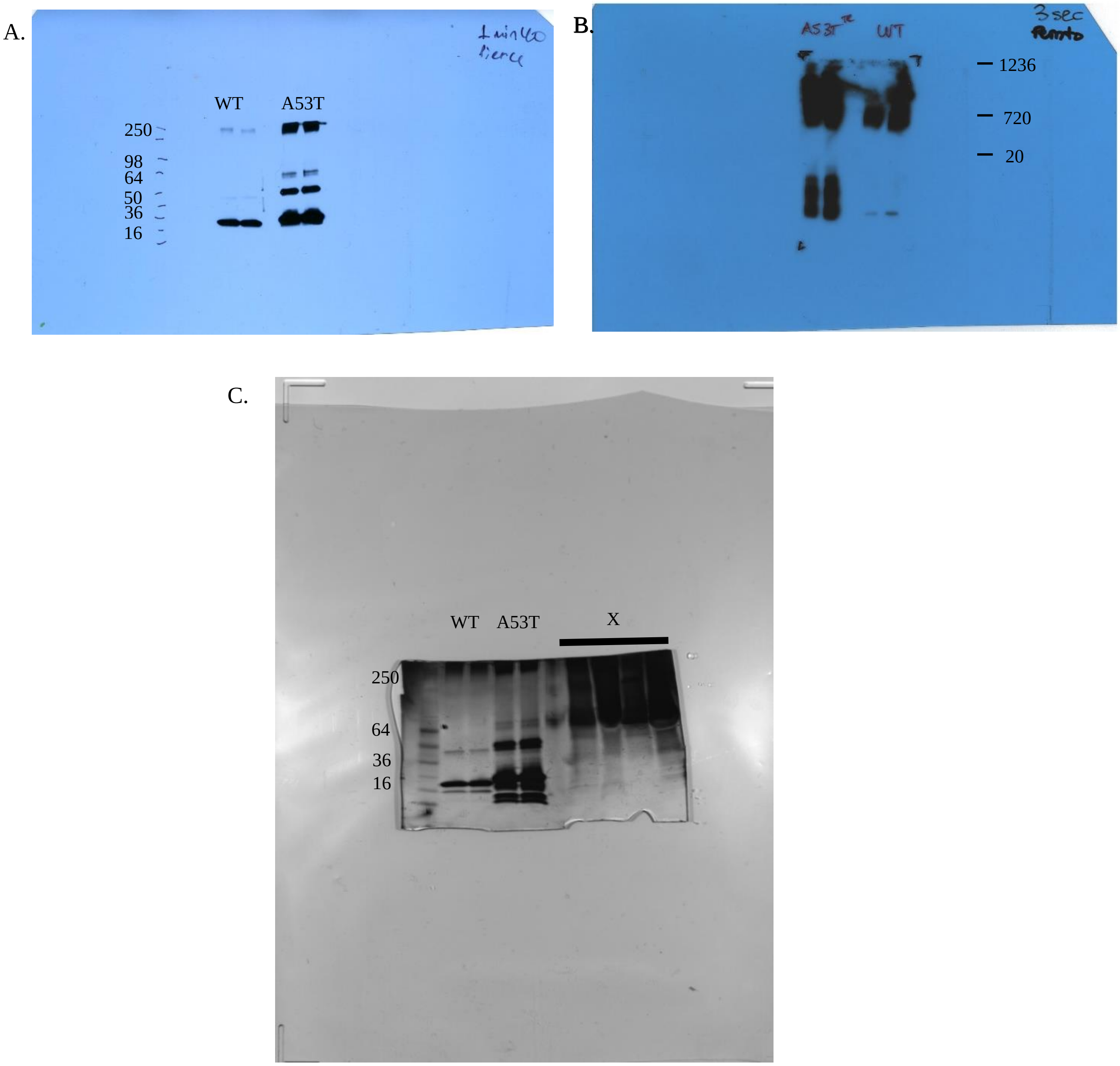

Supplement: Supplementary file 1 [file Image_1.pdf]
